# Supplementary material for: Are carbon emissions trading and green financial instruments synergistic? -Comprehensive quantitative research based on content analysis
Source: PLoS One. 2024 Mar 7;19(3):e0298601. doi: 10.1371/journal.pone.0298601 (PMC10919601; doi:10.1371/journal.pone.0298601)
Supplement: S1 Data — (ZIP) [file pone.0298601.s001.zip › Data/地级市Z_F工作报告.zip!/║┌┴·╜¡/╦τ╗»╩╨/└┤╫╘╣1⁄2╓┌║┼í╛┬φ┐╦╩2╛▌═°í┐_2013.docx]

各位代表:

我代表市人民政府，向大会报告工作，请予审议，并请政协委员提出意见。

一、2012年工作回顾

过去一年，我们紧紧依靠市委的坚强领导和人大、政协的监督支持，按照省委、省政府战略部署，全面落实市委“发展五型经济、建设五个一流、推进六个跨越和十大创新升级，实现中心崛起、富民强市”发展战略，全面超额完成了年初确定的各项任务。

——主要经济指标快速增长。预计国内生产总值达到1058亿元，增长16%；规上工业增加值180亿元，增长29.5 %；固定资产投资550亿元，增长32%；全口径财政收入120亿元，增长22.4%。地方财政收入80亿元，增长29%。公共财政预算收入55亿元，增长31%；城镇居民人均可支配收入13260元，农民人均纯收入9077元，分别增长18.1%和17.6%；粮食总产330亿斤，增长7.8%。万元GDP综合能耗下降3.2%。地方财政收入占GDP的7.6%、税收占财政收入的60.8%、产业投资占固定资产投资的70%、非公有制经济产业投资占产业投资的96%，民生支出占财政总支出的81.4%。各项指标均完成或超额完成省定指标，增速位居全省前列。

——工业实力显著增强。新续建3000万以上产业项目846个，总投资1989.2亿元，新建亿元以上425个。异地项目328个，总投资605亿元。新开工项目数量和投资总额同比双翻番。产业园区完成基础建设投资13.8亿元，新入区项目408个，市开发区升格为国家级经济技术开发区，其它各园区全部进入省级园区行列。61个新建项目建成投产纳入规上企业管理。规上工业用电量20亿千瓦时，增长20%。国家级高新技术企业发展到14户，省级高新技术产品72个。非公经济增加值完成580亿元，增长18%。

——现代化大农业成果丰硕。千公里科技示范带强力牵动，新建标准化农民专业合作社1311个，新建千万元以上现代农机合作社110个。耕地规模经营面积突破1000万亩。落实“节水增粮行动”65.6万亩。综合治理中小河流195公里。植树造林29.1万亩。推广农业先进适用技术32项，玉米大垄双行栽培技术亩增产128公斤、增收200元。新建水稻智能化催芽基地19处。各级农业产业化龙头企业发展到525家。新建规模化养殖场（区）205个，畜牧业增加值增长8.1%。被确定为全国粮食高产创建整市整建制推进试点市和粮食生产先进市标兵。

——城市建设步伐加快。完成城市基础设施投资32.1亿元，同比翻了一番多。“三供两治”14.24亿元，建筑外立面外改造2.76亿元，绿化、广场2.2亿元。新增绿地227.4公顷。房地产开发1018万平方米，其中保障性安居工程261万平方米。县城以上均实现集中供热，9个污水处理厂投入使用，环境保护力度明显加大。绥伊、绥北高速公路如期通车，新建改造城乡公路1608公里。

——新农村建设势头强劲。新建示范村73个，整乡镇推进城镇化规划14个，开工建设7个。改造泥草房6万多户。改建、新建村级组织活动场所514 个，新建文体广场285处121万平方米，农村饮水安全工程2210处，123万人受益。新建续建千万元以上乡镇村项目721个，总投资914.2亿元。乡镇财政总收入28.2亿元，增长77.1%，超千万元乡镇65个。

——现代服务业繁荣活跃。社会消费品零售总额实现345.2亿元，增长16.2%。新续建大商场、大物流等现代服务业产业项目65个，完成投资25亿元。万村千乡市场和新网工程深入推进。进出口贸易额实现1.67亿美元，增长27%；实际利用外资1.51亿美元，增长15%。新增金融服务主体31家，贷款余额突破400亿元，超年度目标100亿元。旅游、信息等产业稳健发展。

——民生和社会事业持续进步。改善民生的50件实事全部完成。城镇新增就业6.76万人，新增公益岗位1.35万个。企业养老保险27万人，城乡低保52.3万人，新农保基本实现全覆盖。新改扩建公办幼儿园132所，全市两基教育工作得到国务院表彰奖励。7个县级高水平医院相继建成，16个乡级卫生院和86个村级卫生所完成改造。新农合参合率99.3%。人口自然增长率达到国家标准。文化体育事业繁荣发展。城乡养老服务和五保供养工作居全省前列。在全省率先启动物价惠民超市。

——各项改革扎实推进。深化市直部门预算改革，化解单位债务1.43亿元，保运转增效益促发展能力明显增强。深化以绩效工资为核心的教育改革，严格遵循政策，精干管理层，强化主干线，分流后勤人员，全市新引进师范本科生694名，分流人员517名，收回高中社会化食堂宿舍4个，清华、北大等一表录取率明显提高。深化医疗卫生分配和基本药物制度改革效果显著，医疗单位业务收入上升26.3%，乡镇卫生院人均医疗费用下降32.8%，市第一医院引进20名研究生。全面清理规范了市属事业单位机构编制，收编、撤销、整合、转变经费形式50家，收回编制551个。深化市区管理体制机制改革，城市秩序、市容市貌、环境卫生明显好转。推进了部分单位运行机制改革，有效解决了厂办大集体、农林“四场”以及永安、津河灌区职工生活保障等历史遗留问题。

——市本级发展建设变化明显。预计市本级全口径财政收入25.7亿元，增长23%。地方财政收入13.1亿元，增长30%，公共财政预算收入7亿元，增长32%。开发区全口径企业税金1.63亿元，增长32.5%。33个亿元项目开工建设，18个投入生产。投资3.5亿元完善了市区市政基础设施，新改建街路23条，完成11条道路排水工程建设，西湖公园、一中过街天桥、高标准公交站亭建成使用。投资3500万元开工建设第三水厂。“一户一表”改造解决了3000多户居民吃水难问题。投资8000万元完善了开发区基础设施。投资1.5亿元建成了调峰热源项目。投资5000万元改造了宝山镇和绥北路部分路段。投资4800万元进行建筑外立面改造22.8万平方米。投资4200万元新增绿化面积100万平方米，90%以上的主次干道实现绿化升级。投资1700万元改建7条街路路灯1250基。投资2000万元添置更新了环卫设备。投资2000万元为市第一医院购置了256CT。新开工保障性安居工程3861套，建设廉租房300套。大唐热电联产项目获国家能源局“路条”。房地产开发秩序、建筑秩序、工程质量以及牌匾规范、占道经营、私接滥建等10项城管执法专项治理成效明显。提高了市区职工和居民大病报销比例和支付限额，干部职工供热、住房公积金、医疗等各项补贴均按省上限兑现。

——政府自身建设不断加强。认真贯彻落实市委各项决策决议，自觉接受人大及其常委会的法律监督、政协的民主监督、公众的社会监督和新闻舆论监督。坚持依法行政，科学民主决策，强化行政监督。制定完善了社会管理、土地征收等领域工作规定，健全了听证、论证、集体决定、结果公示相结合的政务公开机制。公务接待进一步规范，创新开展了政府性支出等多项审计，廉政建设责任制深入落实，公务员队伍作风明显好转，抓落实能力明显增强。

公共节能、民族宗教、外事侨务、妇女儿童、老干部、残疾人、地方志、地震、人防、档案、国防、双拥等工作也取得新进步、新成果。

各位代表！战略决定方向，思路决定出路，措施决定速度，创新决定发展，作风决定实效，民生决定民心！绥化一年来有目共睹的巨大变化，主要是得益于：

——紧紧依靠市委的坚强领导、人大和政协的有效监督，把握正确方向，成就了大发展大跨越的新局面。

——实施发展五型经济、建设五个一流、实现六个跨越、十大升级创新的战略、目标、措施，谱写了大发展大跨越的新篇章。

——深入改革创新，突出主题、贯穿主线，稳中求进、以进为主，不断完善工作推进机制、政策激励机制，迸发了大发展大跨越的新活力。

——发扬求真务实的精神，坚持工作落实是核心，增强执行力是关键，一抓到底，取得了大发展大跨越的新业绩。

——坚持发展成果由人民共享，把改善民生作为加快发展的出发点、落脚点和持久动力，赢得群众广泛理解和普遍支持，凝聚了大发展大跨越的新力量。

这些体会告诉我们，我们的发展完全符合科学发展观，我们一定要认识规律、把握规律、运用规律，倍加珍惜、大力发扬。

在此，我代表市人民政府，向全市人民，向市人大代表、政协委员，向离退休老领导、老同志，向各民主党派、工商联、人民团体和社会各界人士，向驻绥部队指战员、武警官兵和中省市直各部门，向所有参与、支持和关心绥化发展的朋友们，致以最崇高的敬意和最衷心的感谢！

我们清醒地看到，我市经济社会发展总体上仍处于低基数、低起点、低层次阶段，发展质量、收入数量在全省处于靠后位置的状况还没有根本改变，发展仍然是解决我们所有问题的关键。产业、城乡、区域之间发展不协调不平衡，“四化”统筹任务艰巨；城乡居民增收缓慢，建成更高水平小康社会紧张急迫；一些政府工作人员公仆意识、服务水平、落实能力、廉政勤政与人民要求还有一定差距。这些问题，我们必须高度重视，在今后工作中认真解决。

二、2013年工作安排

2013年政府工作的总体要求是：认真贯彻党的十八大和省委经济工作会议、市委三届三次全委（扩大）会议精神，以邓小平理论、“三个代表”重要思想、科学发展观为指导，全面落实省委“八大经济区”、“十大工程”战略，以发展“五型经济”，建设“五个一流”，推进“十项升级创新”，深化“六项重点改革”，实现中心崛起、富民强市目标为统领，深化改革开放，推动升级创新，努力夺取产业项目三年攻坚战的全面胜利，努力实现民生福祉的全面提升，努力保持经济社会重点发展指标的全面跨越增长。

按照“稳中求进”，“进”是核心、是重点、是第一要务的要求，保持较快的发展速度、追求较高的发展质量、取得较好的发展效益、积攒较强的可持续发展后劲，深化改革，升级发展，有力促进财政收入增加、社会潜力增强、群众收益增多，力争2015年地区生产总值、城乡居民收入比全国提前五年、比全省提前两年实现翻一番，2020年翻两番，提前建成更高水平小康社会。按照这一目标，国内生产总值、全口径财政收入、公共财政预算收入、城乡居民人均收入均增长15%以上。到2020年，三次产业结构调整为13：50 ：37，合作农业、现代农业经营面积占耕地比重达到80%，农业机械化率达到99%，工业化率超过50%，实际城镇化率达到70%。

2013年的主要预期目标是：地区生产总值增长16%以上；规上工业增加值增长29%以上；固定资产投资增长30%以上；社会消费品零售总额、进出口贸易额、实际利用外资分别增长15%以上；公共财政预算收入增长18%以上；城镇人均可支配收入和农民人均纯收入分别增长15%以上；万元GDP综合能耗下降3.2%。

（一）深入开展产业项目决胜年活动，着力落实推进招商引资和产业项目、产业园区建设

坚持强工扩总量，围绕加快转变经济发展方式，加速经济结构调整，改造提升传统产业“老字号”、深层次开发资源加工“原字号”、培育壮大战略性新兴产业“新字号”，大上科技支撑型、资源节约型、低碳环保型、税收效益型、产业延伸型、就业拉动型项目，努力提高工业化率，走出一条新型农区工业化发展之路。

做大增量。加快发展必须做大增量，以增量扩总量、调结构、转方式。要加大招商引资力度，深入开展“三冬”活动，坚持项目集中签约新闻发布、分批集中开工。坚持招商引资成功的经验和做法，严把项目准入关，突出高端定位、科学布局、特色发展。重点围绕以合作研发或买断大专院校、科研院所发明专利方式，引进战略投资；围绕退二进三发达城市招商引资，引进产业转移项目；围绕上级有信息、资金、政策、项目单位，帮助和直接招商引资；围绕拉长增粗产业链招商引资；围绕引进高层企业家，通过企业家的影响力、号召力引进项目；围绕带动区域、产业发展，引进战略投资家。努力增速、提质、高效，实现项目总数、项目增量、资金到位率和10亿元以上项目都多于去年，项目质量高于去年的“四多一高”目标。

做活存量。财政持续增收必须壮大企业，关键在做活存量，以存量促增量，延长产业链条。要实施创新驱动发展战略，健全以企业为主体、市场为导向、产学研用紧密结合的技术创新体系，形成利益共同体，使企业真正成为科技创新的主体。支持重点企业建立研发机构，实施知识产权和技术标准战略，促进科技成果应用转化，推动信息化和工业化深度融合。推进企业资产重组，扶持企业上市融资。

做强载体。集约集群发展必须做强做大载体，提升承载能力，大力发展异地经济，提高产业集中度。要完善提升园区规划设计，加大园区基础建设投入，合理规划园区功能布局，促进园区与城区深度融合，提高园区项目承载力、集约集聚力、发展带动力。重点引进科技含量高、投资密度大、产业链长的项目，弥补产业链缺失环节；引进先进技术和装备，促进发展具有自主知识产权和核心技术的高端产业；引进新兴、战略性产业项目，培育新的增长点；引进金融、现代物流、总部经济等为代表的高端服务业项目，力促二、三产业协调发展；引进高新技术和高端服务业，提高城市资源聚集力和功能辐射力；注重第一产业类项目延长做强，吃干榨净；有限制有选择地引进因市场发育而形成的竞争类项目。

做优质量。提高回报率必须做优质量，强化园区和企业管理，向管理要效益。要突出骨干企业，加强经济运行调控。重点处理好提高速度与保证质量、规上企业与小微企业、做大增量与做活存量、园区建设与招商质量、项目建设与服务要素、项目运营与上市准备、市场销售与地工产品采购、企业融资与金融信贷、规范管理与企业培训、加快发展与安全生产等十个方面关系。

做大市场。提高活力必须做大市场，着眼扩大内需，努力扩大市场份额。实施品牌战略，支持扶持企业创建打造名牌产品。加强市场营销，创新商业模式，提高产品市场竞争力、占有率。加强农副产品与销区连接，建立平台，完善网络，打造产销对接绿色通道。

做壮队伍。经济微观基础是企业，企业发展关键在于提高企业家队伍素质。抓好企业家队伍培育至关重要。要加强对企业高管人员的培训引进，加大对贡献突出的企业家宣传奖励力度。注重企业文化建设，增强企业软实力。

（二）提升城市整体功能, 着力落实推进城市型城市群经济

要坚持建城提品位，围绕环境让城市更宜居、城市让生活更美好、发展让人民更幸福、文化让绥化更和谐，规划城市、建设城市、管理城市、经营城市、发展城市统筹一体推进，努力建设北方大平原生态型、园林式、现代化城市。

高标准规划城市。城镇建设一个时期始终是我们要牢牢把握的建设重点。坚持先规划后建设，按照政府主导、专家领衔、部门合作、公众参与、规委会审定原则，认真抓好各项规划。加快辟建新城区，适度改造旧城区，增加公共服务功能，建设产业园区，实现产城一体化。完成城市总体规划和新区规划编制工作，增强规划的执行力和约束力，坚持容积率标准，加强规划设计、研究机构建设，认真管理好城市基础设施档案。

高质量建设城市。设计是城市特色的前提和保证。要加强城市整体风格、功能布局以及区域景观和单体建筑设计，严格工程质量。突出“一水二路三公益”的财政投入重点和建设顺序，统筹安排城市各项要素，体现投资价值，提升城市功能，强化配套服务，增加经营效益，达到集约、智能、低碳、绿色、安全、经济、适用、美观的要求。突出推进“三供三治”、城市路网等基础设施建设，配套跟进教育、文化、金融、医疗、商贸等公共服务设施。

精细化管理城市。坚持“政府主导、社会协同、群众参与、市场化推进”，建立长效机制，促进城市管理制度化、数字化、机械化、社会化、市场化。完善城市保洁、园林绿化、交通秩序、客运秩序、经营秩序等方面管理标准。提升市民文明素质。加强对历史建筑和树木的保护，着力打造具有北方特色的大平原城市文化。

高效益经营城市。坚持量力而行，尽力而为，科学经营城市，有序发展城市。经营好土地，拆平建楼、以楼换地、以地经营、以城养城，形成健康有序、良性循环的土地市场。注重打造经济圈，科学设计整体布局，积极培育商贸、金融、文化、饮食、物流中心等板块。突出城市功能热点，强化功能拉动，增强示范效应，吸引游离资本。掌控供求关系，有序调控房地产、商贸中心、娱乐场所等城市产出品供给，保证健康有序发展。进一步完善城市投融资平台，改革做强城投公司。

打造生态城市。强化生态文明建设，突出城市特色、彰显城市品位。抓好城市绿化，重点打造带状、块状、片状林，高中低、多品种推进。新建小区绿化用地不低于总用地的30%，老旧小区改造占到25%。力争10万人以上城区每人平均1棵胸径10公分的树，10万人以下每人平均2棵。加大坑塘弃地治理，打造园林景观。加强裸土治理，县级以上建成区内要基本实现绿化或硬化。全面加强环境保护，推进农村环境综合整治，抓好热力企业脱硫脱硝、城市烟尘噪声、汽车尾气污染治理，完善污水处理厂功能，确保污染减排全部达标。

（三）构建新型农业经营体系，着力落实推进现代化大农业建设

坚持抓农固基础，提升农业素质，大农机、大水利、大科技、大合作组合推进，努力创建全国现代化大农业示范区，建设生态农业、科技农业、标准农业、设施农业、效益农业、产业农业。以工业化引领农业发展、城镇化引领农村发展、市场化引领农民发展，推进农民持续快速增收、农业持续高产稳产、农村全面同步小康。

强化农业基础设施建设。深入开展国家粮食高产创建整市整建制推进试点工作，确保粮食稳产增产。强化农田水利、农业科技推广、农机物质装备等农业基础设施建设，新建水稻智能化催芽基地21个，新增覆盖面积160万亩。加强生态农业建设，抓好水土流失综合治理，完成节水增粮74万亩，造林绿化15万亩。

提升千公里科技示范带。推进扩面、增容、提质，上标准、扩规模、增效益。拉长战线，扩增到10条主要公路沿线，延伸到2000公里，同时向各支线公路延伸扩展。加密布点，示范园区数量达到528个。扩充内容，将原有5种示范园区扩充到10种，向复合型、全方位发展。提高标准，全程全面植入标准化，同步提高生产标准与生态标准、作业标准与建设标准。放大效应，强化种植业、养殖业、加工业等农业综合技术集成创新和新农村、林业生态等方面的示范带动作用，增加会展、培训、窗口等功能。

加快发展合作农业。坚持农村基本经营制度，保证土地所有权不变，以合作农业为载体，农机合作社为牵动，加快推进土地流转。重点发展农机合作社，主推农机拥有者领办合作社、农民入股成立农机合作社、农机合作社租种土地、代种代耕“四种形式”。全市新建农民专业合作社800家，水旱田农机合作社100家。力争三分之一以上的耕地流转规模经营，三分之一的农民从土地中分离出来，三分之一的农民有新就业。

推进农业产业化升级。突出创新思维、品牌意识、科技手段，从绥化实际出发，加快发展循环经济，突出种养加综合开发，资源循环利用。重点抓好粮食生产、饲料加工供给、秸杆及副产品加工、种子生产加工、产业包装生产、餐桌服务、农产品物流销售、信息化服务等产业线。做活农产品精深加工，壮大农产品加工企业集群，实现农副产品吃干榨净、产销两旺。

促进农民多元增收。全面建成小康社会农民是难点、是重点。没有农民小康，就没有全面小康。我市有420万农民，任务更为艰巨。农民收入是核心、是关键，必须多措并举，使农民有效益、不吃亏、得实惠，促进农民快速稳定持续增收。要积极引导农民从优化种养结构、错季销售、精深加工、终端市场等各个环节中增加收入。优化种植业结构调整，减大豆、增玉米水稻、上经作，大力发展棚室蔬菜、林下经济、食用菌生产，每个县（市、区）都要建成一个食用菌种植示范基地。要突出发展现代畜牧业，科学规划，一业为主，多业并举，突出重点，体现特色，建成黑龙江的养殖大市、标准大市、加工大市、效益大市和畜牧安全大市。坚持规模化发展，建好畜牧示范园区，畜牧业规模化养殖比重达到60%以上。继续推进小规模、大群体，扩大总体数量，吸引养殖户进入园区。提升畜牧产业化，扶持培育壮大龙头企业，增强拉动能力。完善服务体系，提供全程配套服务。要推进农村劳动力有组织转移，加强职业技能培训和农民工权益保障，大力发展农村第三产业，积极发展小微企业，支持农民创业增收。

（四）突出整乡镇推进城镇化，着力落实推进社会主义新农村建设

城镇化是我们现代化建设面临的历史性任务，是经济增长和结构调整的重要引擎。要突出建设重点，以“撤屯、减村、并乡、建城”为基本路径，着眼城乡发展一体化，提高城镇化率。坚持规划在先，产城融合，试点带动，梯次推进，特色发展，做实做强产业，集中集约建设，推动产业结构、就业方式、人居环境、社会保障等一系列由乡到城的根本跨越，建设宜居乐业新城镇新农村。

优化总体布局。按照“以城镇建设为重点，整乡镇推进为试点，适度建设中心村”的思路，加快建设中心镇、中心社区，实现人口集聚、产业集中、土地集约发展。完善村镇整体建设规划，坚持与城市发展总体规划、园区发展规划紧密衔接。统筹道路、水、电等各类基础设施建设，防止重复建设和投入浪费。

加快推进步伐。突出抓好整乡镇推进城镇化，带动整村推进。在坚持新农村建设“市场开发、梯度推进、旧村改造、企业牵动、乡村主导”五种模式的基础上，深入实施“宅基地开发置换、市场开发置换、政府主导开发置换、耕地等农牧资源由加工龙头企业置换、补偿土地增减挂钩开发置换”五种整镇化推进模式。每个县至少抓好2个整乡镇推进，3-5年建成一个，5-7年建成一个，带动100个示范村。全市城镇村建设面积达到1000万平方米。

做强产业支撑。坚持产城一体，统筹规划产业发展，确保搬得进、住得起、富得了。做强农业产业，提高土地节约集约利用水平，有序推进农业转移人口市民化、产业工人化、土地收益股东化。大力发展乡镇村经济，招商上项，做大总量，以工补农。政府主导城镇开发，加快发展城镇商贸业，促进农民就近就地就业。

强化功能配套。加快公共设施向建制镇、中心乡镇延伸，公共服务向农村覆盖。着力完善重点城镇功能，加强道路、公园、广场等基础配套建设，创优人居环境。建立健全各类便民服务机构，逐步完善城镇社会保障和救助体系，加强教育、医疗、文化等社会事业建设，推进中心村社区化管理。

（五）挖掘扩大潜力，着力落实推进乡镇村经济

以园区为载体、以异地经济为实现形式，充分发挥乡镇村行政单元活力，壮大乡镇村经济规模，提升乡镇村整体实力。乡镇财政收入和村集体收入分别增长30%以上。

壮大乡镇村力量。强化干部队伍建设，不断增强发展能力。落实落靠村社区干部各项政策待遇。严格考核奖惩机制，激发干事创业热情。建立科学的人才流动机制和多元化人才引入机制，发挥好大学生村官作用，培养农村基层人才队伍，把农村基层优秀人才吸纳进“两委”。

增强乡镇村实力。县（市、区）统筹园区建设，统筹项目摆布，乡镇村项目以县级园区落地为主，优化结构布局，提高乡镇村项目质量。坚持实体财政或“双轨制”运行，努力增加乡镇财政收入，保障乡镇村发展活力。发展壮大百姓经济、基地经济、畜牧经济，实现乡镇村经济多元发展。组织开展好强村结对帮扶弱村活动，提高贫困村发展建设能力。全市财政收入超千万元乡镇增长20%以上。

强化乡镇村管理。切实加强乡村集体资金、资产、资源管理，严格完善“三资”管理制度，深入推行村级账务公开。乡村两级所得收入，用于改善民生和发展乡镇村经济。

（六）创造集约优势，着力落实推进异地经济

强化异地园区建设，创新异地经济发展模式，完善利益分配机制，更大范围优势共享。

科学功能布局。实行差异化发展，突出比较优势，推进区域分工协作，增强产业竞争力，推进项目合理落地。建设域外企业经济专区。

强化政策引带。完善激励政策，健全绥化内部异地园区政策，吸引项目落户园区。重视外部创新资源的获取，引导企业与大专院校科研院所建立合作网络。建立异地园区内部协调合作机制，推进入园“异地企业”与“本地企业”快速融合。

合理配置发展要素。建立城乡间、区域间的资源、人才以及各种发展要素共享机制，完善异地项目集中区管理机构建设，发挥好异地园区产业发展规划、基础设施建设、环境保护、公共服务等服务职能。

统筹区域发展。推进优势地区强势发展，劣势地区借势发展。统一全市招商政策，实行区域政策一体化，防止无序竞争。拓展异地经济合作范围，创建立足市域、发展省内、面向全国的共赢合作发展格局，建设更多的省内外合作共建产业园区。

（七）提高融入能力，着力落实推进都市圈经济

坚持活贸兴产业，围绕大都市、促进大合作，不断激发全市内在发展活力，提高各项产业关联度、外向度，在更宽领域、更高层次推动都市圈经济快速发展。

主动承接产业转移。瞄准哈大齐重点城市，大力发展同体型经济、协作型经济、吃配型经济、服务型经济和城郊型经济。肇东、兰西重点做大现代服务业，建好五站、东发新城和哈北新城。安达重点融入大庆石油石化产业，建好北湖新城和卧里屯小镇。

突出发展服务业。抓好大型商贸节点项目筛选工作，在市场体系建设、出口产品研发、现代物流发展等方面，用好用足国家拉动内需政策。巩固提升传统市场，积极开拓新兴市场，开展服务贸易。突出发展面向生产的现代服务业。打造旅游节点城市，把我市旅游定位在短期假日近距离休闲游，大力发展乡村风情游、农业观光游、自然生态游、郊区特色游。

加快发展金融业。全面优化金融生态环境，在重点产业、重点领域和扶持企业发展上加大信贷投放力度。支持域内银行机构恢复或增设县级网点，抓好小额贷款公司，鼓励引导企业探索多元化融资模式。规范发展保险业，扩大农业政策保险覆盖面，加快发展证券服务业。全市金融机构贷款余额力争增长15%以上。

提高开放型经济水平。深入实施走出去战略，扩大对外开放，更好的开发利用两种资源两个市场。通过升级招商引资、人才技术合作、向上争取、搞好资本运作和拓展金融合作开放空间，以及通过优势资源、优势市场、优势环境推进开放，参与拓展对俄和东欧各地区的开放合作。大力培育出口创汇龙头企业，壮大进出口群体，挖掘新的贸易增长点，推动贸易多元化发展，提高经济发展外向度。

（八）加大固定资产投资力度，着力落实推进基础设施和重大工程项目建设

围绕国家政策导向，加快投资建设一批重大基础设施、社会民生工程项目，提升发展实力，增加发展后劲。

强化项目生成。抓住国家继续增加城镇化、农林水利、公路、“棚草”改造、千亿斤粮食产能、生态环保、公共服务等重大基础设施和民生工程投入的政策机遇，围绕城市基础设施、农业农村基础设施和社会事业基础设施建设，有计划、有重点的谋划实施一批重大项目。

加大资金投入。积极向上争取，充分发挥政府投资带动作用，用好城投债资金，撬动社会资本投向重点领域基础设施建设。全力推进铁路、公路、水利、生态、环境保护等重点建设工程。全市计划启动大唐热电联产、城区供水、道路、供热管网、建筑外立面节能改造等城市基础设施建设项目5项，总投资43.62亿元；绥化至大庆高速公路、原哈黑路（兰西至明水段）改造升级、哈齐城际铁路（肇东、安达段）、集中连片特困地区交通扶贫（明水、青冈、望奎、兰西）、国省道改造（望奎至四方台互通段、老哈伊路宝山至绥化开发区段等）、绥满公路哈肇界至肇东段等交通基础设施建设项目6项，总投资217.2亿元；“节水增粮”工程、农机化工程、土地整治工程、宝山现代农业示范园等农业农村基础设施建设项目4项，总投资52.3亿元。

加强项目监管。严格工程质量和安全监管,强化安全生产、环境保护三同时，对重点项目适时实地监控，确保重大项目规范实施。严格工程验收标准，按照法律法规和程序制度验收，确保工程合格。严格项目资金监管，确保项目建设资金专款专帐专户，规范透明。

（九）加强社会建设，着力落实推进更高水平的民生改善保障

加强和创新社会管理，加快健全基本公共服务体系，保障和改善民生，在学有所教、劳有所得、病有所医、老有所养、住有所居等方面取得新进展，推动和谐社会建设。

提高社会保障水平。深入实施就业优先战略，抓好重点群体就业，全市新增就业岗位6万个，下岗人员再就业2万人。继续推进“五险”统筹扩面，实现应保尽保。全面提高城乡居民最低生活保障和五保供养标准，完善物价上涨与低收入群体价格联动机制。继续推进中心敬老院和五保家园建设，扶持民办养老机构加快发展，推动居家养老服务全面开展。农村五保对象集中供养率达到65%以上。推进城乡社区达标升级，80%的街道社区建成社区服务站，40%的农村社区建成服务中心。加强乡镇公共服务中心建设，提供社区保障经费，尽力满足服务群众、救助困难群体的基本需求。

提高文化建设水平。加快“文化强市”建设，丰富人民精神文化生活。加快公共文化设施建设，抓好文体惠民工程。开展好广场文化、农村送戏、送图书、送电影、送辅导等活动。加快文化产业发展，培育新兴文化产业，壮大重点文化企业，提升基础较好、特色明显的文化产品规模、档次和影响力。

提高社会事业水平。坚持教育优先发展，健全教育投入保障机制。深入实施素质教育，着力提高教育质量，合理配置教育资源，抓好教师补充培训，促进各级各类教育均衡协调发展。加强校车、小饭桌管理。加强师德师风建设，严肃治理乱办班、乱补课、乱订教辅资料，努力办好人民满意的教育。完善城乡医疗卫生服务体系，加强基础建设，完善技术装备，新改扩建乡镇卫生院10个、标准化农村社区卫生所60个。全市新农合基本全覆盖。落实国家基本药物制度，落靠城乡人均25元公共卫生基本服务费。规范民营医院发展，清理非法个体经营诊所。积极开展全民健身活动，继续抓好人口“惠家工程”，人口自然增长率控制在4‰以内。

提高社会管理水平。坚持“民生优先、服务为先、基层在先”，加强和创新社会管理。增强城乡社区服务功能，完善网格化、立体化社会安全稳定防控体系。提高信访工作科学化水平，维护群众合理合法诉求，解决好群众关心的热点焦点问题。加强应急管理，强化安全生产，突出抓好重点行业和重点领域安全隐患排查整治,杜绝重特大事故发生。开展文化娱乐场所集中整治，突出校园周边环境综合治理，加强网络信息安全管理。坚决深入开展打黑除恶专项斗争，严厉打击黑恶势力和各种非法邪教组织。严查严打黄、赌、毒等各类违法犯罪活动。突出打击垄断资源等各类经济领域违法犯罪。

同时，抓好公共机构节能、防震减灾、外事侨务、妇女儿童、民族宗教、老干部、残疾人、地方志、国防、双拥、人防、老龄、档案等工作。

（十）以人为本执政为民，着力落实推进政府自身建设

把作风作为民心所向，把提高执行力和优化发展环境作为加强政府自身建设的突破口，创新服务，力促发展。

加强学习，提高能力。深入学习贯彻十八大精神，切实提高政策执行力、改革创新力、科学发展力。加强和改进调研，走进基层群众，走进困难矛盾，了解真实情况，解决实际问题。勇于实践，敢于负责，提高各级干部干事创业能力、破解难题能力、促进和谐能力。

依法行政,廉洁从政。主动自觉接受人大、政协的依法监督和民主监督，广泛接受媒体监督和社会监督。深入推行政务公开，完善行政决策机制。认真落实党风廉政建设责任制，强化权力运行监管，厉行勤俭节约。加大对土地出让、政府采购、工程招投标、国有资产处置和大额资金使用等重点领域的行政监察和审计监督，严肃查处各类违法违纪案件，做到干部清正、政府清廉、政治清明。

转变作风，创新落实。坚持工作落实是核心，增强执行力是关键。认真执行中央改进工作作风、密切联系群众的八项规定，坚持实干兴邦、实干富民，求质重效、立说立行。改进会风文风，规范政务接待，简化公务活动。强化效能监察，优化经济发展环境，坚决治理懒政、怠政、惰政。全面整合行政服务资源，清理规范行政事业性收费。加强社会诚信体系建设，推进政务诚信、市场诚信、个人诚信。

深化改革，激发活力。重点推进合作农业为载体的现代化大农业、城镇化为重点的新农村建设、产业园区经济、异地经济、乡镇村经济、都市圈和城市型城市群经济等六个重点领域具有重大深远意义的改革深化和发展升级。同时，继续推进行政审批制度改革，清权确权，精简审批。推进部门预算制度改革，增强预算刚性化，实现“三公”经费零增长。推进城市管理机制改革，实现规范化、高效化、科学化管理。积极稳妥推进事业单位分类改革，深入实施教育卫生绩效工资改革。推进市经济开发区管理机制改革，增强发展活力。

全面经营，全程管理。坚持投入讲回报、资金讲节约、成本讲预算、经营讲实效、管理讲规范。行政手段和市场手段合理组合，经营管理好一切优势资产和资源，建立统一的国有资产管理平台，加大资源、人员、收入、支出等资产资源的管理力度。推进开源节流，实现全面经营增收、全程管理增效。

三、全力抓好市本级发展

在大力推进全市经济社会加快发展的同时，更加突出加快市本级发展，增速、提质、高效，把市本级尽快打造成为全市园区建设的高新区、新城建设的样板区、现代服务业的示范区、民生事业的模范区。

——增强开发区实力。深化市直部门招商引资激励机制，充分调动全体人员招商引资、服务企业、管理指导的积极性，加大招商引资和产业项目建设力度。投入1.2亿元完善开发区基础设施，建设亿元以上项目30个，全口径企业税金增长30%以上。

——加大城市建设力度。把新区建设作为2013年乃至今后一个时期城市工作的重点。全力建设新城区，适度改造老城区，增加城市功能。坚持高标准规划，高质量建设，高水平管理，对未来城市发展起到示范引领作用。突出打造一区两城（一个主城区，宝山镇、东富乡两个卫星城）。开发区完成宝山镇卫星城规划设计，北林区完成东富乡卫星城规划设计，并力争启动建设。市区房地产开发150万平方米，改造3处棚户区，棚改面积80万平方米，新区建设70万平方米。投入5亿元，开工建设道路、供水、公园、广场、学校、医院等公共公益基础设施。更加注重精细化、科学化、规范化、法制化，消除城市管理的死角死面，提高城市管理水平。

——提升城市服务业层次。加快发展物流业，辟建一处10万平方米现代物流园。加快汽配城建设，发展好吃配经济。繁荣规范农贸市场，突出现代服务业设施建设，辟建大型商贸综合体。规范各类中介服务机构，理顺经营秩序。充分发挥现代服务业对拉动经济发展、提升群众福祉、满足生产需要的综合效应。

——加快社会事业建设。创建全省一流的教育、卫生等社会事业，在全市起到示范带动作用。继续深化完善以绩效工资改革为重点的教育管理机制改革，加强教育工作者的引进、培训和后勤管理人员的分流，强化一线、精干后勤，提高教育教学质量。继续深化完善以分配制度改革为重点的医院管理机制改革，抓好高端医务工作者的引进、医护人员培训和后勤服务社会化分流，全面提高诊疗服务水平，扩大区域知名度和影响力。开展好广场文化演出活动，坚持体育馆、图书馆、文化馆每天向公众免费开放，不断增强市民文化素质和文明程度，提高文化软实力。

——提高财税管理水平。开源节流、增收节支同步推进，共同发展。完善制度机制建设，科学税收征管，强化非税收入管理，做到应收尽收。加强国有资产管理，加大经营力度，确保保值增值。增强节约意识，强化支出监管，提高制度化、常态化管理水平，率先实现“三公”经费零增长，发挥财政资金保运转促发展的作用，促进公共财政预算收入持续增长。

各位代表，为民用权、替民谋利，万事靠干、重在落实。市本级要率先建设更高水平的民生，就必须沉下心、沉下身、沉下力，每年都干成几件老百姓看得见、摸得着的实事好事。2013年，我们将全力办好50件民生实事。

城市建设： 1、投资5亿元完成新区道路、绿化、广场、社区、学校等基础设施和公益设施建设。2、建设保障性住房3000套，公租房100套。3、续建森林公园二期工程。4、在市区新建三处植物园。5、投资街路绿化2000万元。6、改造人民公园、火车站、圈树三处公园广场，新建4.3万平方米、1.4万平方米两处广场。7、新建路灯1102基。8、规划建设一处10万平方米大型商业综合体。9、规划开工大唐热电联产项目。10、开工建设垃圾发电项目。11、投资2000万元建设一座多普勒气象雷达站。12、新建一处2.2万平方米地下停车场。13、开通康庄路、开发区公交。14、推进加快雨污分离工程和污水再生水利用工程建设。15、改造第一、第二水厂，完成第三自来水厂建设，完成2个小区供水改造，提高供水标准。16、完成建筑外立面改造20万平方米。17、打通绥巴路、黄河路至开发区道路，城区至科研路5条道路全部联接贯通。18、投资5000万元，完成康庄路沥青混凝土罩面。19、结合环城水系建设，完成南小沟污水排放改造的规划、设计和立项。20、投入1.2亿元加强开发区基础设施建设。

社会保障：21、投资3000万元的老年供养中心建成并投入使用。22、投放200万元，建立见义勇为奖励资金、贫困学生助学资金、劳模解困救助资金、扶贫解困救助资金、突发事件应急安置资金。23、低保标准从月人均265元提高到330元。24、新农合人均筹资标准由290元提高到350元。25、新增企业养老保险2万人，发放失业保险金167万元。26、发放小额贷款4000万元。27、投放价调基金220万元，用于救助困难群体和低收入家庭。28、建立市工会困难职工帮扶中心。29、“一帮一”4037名被帮扶对象全部脱贫。30、按国家政策提高工资和津补贴标准，及时足额上限兑现。

公益事业：31、新建1所公办幼儿园。32、在新区新建1所建筑面积6000平方米的小学。33、在新区新建1处6000平方米综合体育馆。34、市直属学校招聘优秀师范类毕业生96名。35、完成一中操场回购，为一中、二中新建塑胶跑道各一处。36、完成七中校园搬迁规划和前期准备工作。37、投入2000万元为第一医院购置核磁机一台。市属各医院共出资2000万元购置大型医疗设备。38、完成人民医院扩建改造并投入使用。39、新建1万平方米妇幼保健院住院楼。40、依托科技馆建设绥化特色文化艺术馆。41、投资2亿元新建1座大型长途客运站。

社会管理：42、新建1处武警支队官兵营房。43、投入1000万元强化消防设备设施建设。44、完成市精神病医院建设并投入使用。45、建设标准化社区4处。46、完成城区7个派出所建设。47、依托“天眼工程”，整合应急、信访、交通、治安、城管等各方信息、监控资源，努力建设数字城市。48、消除物业弃管小区，规范发展物业公司，成立由物业管理部门牵头，人大代表、政协委员、居民代表和新闻媒体参加的物业等级评定委员会，公开评定标准，逐一对物业等级阳光评定。49、统一规范交通信号灯信号，整顿规范交通秩序，加强公车管理，出台出租车管理规定。50、清理规范婚姻、就业、殡葬等服务性收费。

各位代表！绥化正处在改革深化、发展升级、经济转型、民生提升的全新历史起点上。让我们在市委的坚强领导下，深入贯彻十八大精神, 着力落实发展“五型经济”、建设“五个一流”、推进“十项升级创新”、深化“六项重点改革”等战略思路，乘势而上，强势而进，改革创新，狠抓落实，为强力推进绥化经济社会大发展大跨越，实现中心崛起、富民强市，提前建成更高水平小康社会而努力奋斗！
